# Improving NKCC1 Function Increases the Excitability of DRG Neurons Exacerbating Pain Induced After TRPV1 Activation of Primary Sensory Neurons

**DOI:** 10.3389/fncel.2021.665596

**Published:** 2021-05-25

**Authors:** Shi-Yu Deng, Xue-Chun Tang, Yue-Chen Chang, Zhen-Zhen Xu, Qin-Yi Chen, Nan Cao, Liang-Jing-Yuan Kong, Yang Wang, Ke-Tao Ma, Li Li, Jun-Qiang Si

**Affiliations:** ^1^The Key Laboratory of Xinjiang Endemic and Ethnic Diseases, Department of Physiology, Shihezi University Medical College, Shihezi, China; ^2^Department of Anesthesia, The First Affiliated Hospital of Guangzhou Medical University, Guangzhou, China; ^3^NHC Key Laboratory of Prevention and Treatment of Central Asia High Incidence Diseases, First Affiliated Hospital, School of Medicine, Shihezi University, Shihezi, China; ^4^Department of Cardiology, First Affiliated Hospital of Shihezi University, Shihezi, China; ^5^Medical Teaching Experimental Center, Shihezi University Medical College, Shihezi, China; ^6^Department of Physiology, Medical College of Jiaxing University, Jiaxing, China; ^7^Department of Anesthesiology, Xiangyang Central Hospital, Xiangyang Central Hospital, China; ^8^Department of Anesthesiology, Institute of Anesthesiology and Critical Care Medicine, Union Hospital, Tongji Medical College, Huazhong University of Science and Technology, Wuhan, China

**Keywords:** DRG, NKCC1, TRPV1, capsaicin, pain

## Abstract

**Background** Our aim was to investigate the effects of the protein expression and the function of sodium, potassium, and chloride co-transporter (NKCC1) in the dorsal root ganglion (DRG) after activation of transient receptor potential vanilloid 1 receptor (TRPV1) in capsaicin-induced acute inflammatory pain and the possible mechanism of action.

**Methods** Male Sprague–Dawley rats were randomly divided into control, capsaicin, and inhibitor groups. The expression and distribution of TRPV1 and NKCC1 in rat DRG were observed by immunofluorescence. Thermal radiation and acetone test were used to detect the pain threshold of heat and cold noxious stimulation in each group. The expressions of NKCC1 mRNA, NKCC1 protein, and p-NKCC1 in the DRG were detected by PCR and western blotting (WB). Patch clamp and chloride fluorescent probe were used to observe the changes of GABA activation current and intracellular chloride concentration. After intrathecal injection of protein kinase C (PKC) inhibitor (GF109203X) or MEK/extracellular signal-regulated kinase (ERK) inhibitor (U0126), the behavioral changes and the expression of NKCC1 and p-ERK protein in L_4__–__6_ DRG were observed.

**Result:** TRPV1 and NKCC1 were co-expressed in the DRG. Compared with the control group, the immunofluorescence intensity of NKCC1 and p-NKCC1 in the capsaicin group was significantly higher, and the expression of NKCC1 in the nuclear membrane was significantly higher than that in the control group. The expression of NKCC1 mRNA and protein of NKCC1 and p-NKCC1 in the capsaicin group were higher than those in the control group. After capsaicin injection, GF109203X inhibited the protein expression of NKCC1 and p-ERK, while U0126 inhibited the protein expression of NKCC1. In the capsaicin group, paw withdrawal thermal latency (WTL) was decreased, while cold withdrawal latency (CWL) was prolonged. Bumetanide, GF109203X, or U0126 could reverse the effect. GABA activation current significantly increased in the DRG cells of the capsaicin group, which could be reversed by bumetanide. The concentration of chloride in the DRG cells of the capsaicin group increased, but decreased after bumetanide, GF109203X, and U0126 were administered.

**Conclusion** Activation of TRPV1 by exogenous agonists can increase the expression and function of NKCC1 protein in DRG, which is mediated by activation of PKC/p-ERK signaling pathway. These results suggest that DRG NKCC1 may participate in the inflammatory pain induced by TRPV1.

## Introduction

At present, pain is still a major problem that troubles mankind in the world because of its complex and diverse production mechanisms (Kuner and Kuner, 2021). Nerve damage, inflammation, and post-operation are the main causes of pain (Xu et al., 2019; Lin et al., 2020; Shepherd and Grivell, 2020). The dorsal root ganglion (DRG), a bridge in the process of peripheral and central sensitization, regulates and transmits nociceptive sensation. The change in ions is the main cause of spontaneous cell discharge that leads to abnormal pain (Theile et al., 2016), and it is gradually receiving attention. Among them, the change of anion has a special effect on the production of pain. When the chloride channel is activated in primary sensory neurons, an intracellular hyperchloric state may result in chloride ion efflux and depolarization (Mao et al., 2012). Studies have found that an increase in sodium, potassium, and chloride co-transporter (NKCC1) in the DRG could trigger pain in a neuropathic pain model in rats. It may also increase the concentration of intracellular chlorine ions, thereby triggering dorsal root reflex (DRR) (Tan et al., 2020). Therefore, regulating the expression of NKCC1 may be another direction for the treatment of pain. The studies demonstrated that NKCC1 might play an important role in inflammatory and tissue damage pain (Galan and Cervero, 2005; Valencia-de Ita et al., 2006). Pitcher et al. (2007) show that spinal NKCC1 and transient receptor potential vanilloid 1 receptor (TRPV1) are critical for referred allodynia mediated by a painful visceral stimulus. Moreover, they suggest that endogenous TRPV1 agonists, released in the CNS in painful conditions, might stimulate TRPV1 receptors on primary afferents that, in turn, play a role in increasing NKCC1 activity leading to allodynia. The time-dependent interactions are also studied in this research (Pitcher et al., 2007). In addition, other studies have shown that after capsaicin was injected in the colon, the expression of p-NKCC1 rapidly (10 min) increased in the spinal dorsal horn of the corresponding segment and NKCC1 increased by 50% in the spinal cytoplasmic membrane of the lumbosacral spinal cord 90 and 180 min after injection of capsaicin in the colon (Delpire and Mount, 2002; Pitcher et al., 2013). These findings indicated that the activation of TRPV1 could regulate the change in NKCC1.

Studies have also shown that protein kinase C (PKC) is involved in regulating the extracellular signal-regulated kinase (ERK) pathway and activating transcription factors. In the latest research reports, ginsenoside Rh3 inhibits skin damage from ultraviolet light by inhibiting PKCδ and ERK phosphorylation in SP-1 keratinocytes (Park et al., 2020). PKCδ regulates astrocyte-swelling diseases caused by ammonia, including hepatic encephalopathy, by activating Src and downstream epidermal growth factor (EGF) receptor/ERK signals (Jia et al., 2020). In addition, the activation of TRPV1 in the lens epithelium leads to multiple calcium-dependent signaling responses, including the activation of ERK1/2; in addition, it can also activate mechanosensitive transporters (Shahidullah et al., 2018). However, there is no report about how TRPV1 regulates the changes of NKCC1 in the process of inducing inflammatory pain. The aim of this study was to elucidate the role of NKCC1 in DRG neurons after acute inflammation pain induced by TRPV1 activation.

## Materials and Methods

### Drug

Capsaicin (TRPV1 agonist; Sigma, United States) was dissolved in Tween-80 (10%), ethanol (10%), and phosphate-buffered saline (PBS). The ultimate concentration of Tween-80 and ethanol neither exceeded 0.1% nor caused any observable effects (Rossato et al., 2018). Bumetanide (NKCC1 inhibitor; Sigma, United States) was dissolved in dimethylsulfoxide (DMSO) and artificial cerebrospinal fluid (aCSF) (1.3 mmol CaCl_2_ 2H_2_O, 2.6 mmol KCl, 0.9 mmol MgCl_2_, 21 mmol NaHCO_3_, 2.5 mmol Na_2_HPO_4_ 7H_2_O, 125 mmol NaCl, and 3.5 mmol glucose; pH 7.2–7.4). U0126 (ERK inhibitor; Sigma, United States) and GF109203X (PKC inhibitor; APExBIO, United States) were dissolved in DMSO and saline, respectively.

### Animals

A total of 200 male Sprague–Dawley rats (Payrits et al., 2017) (10–12 weeks old, 180–250 g) were acquired from the Animal Experimental Center of Xinjiang Medical University (Urumqi, China; license number: SCXK New 2003-0001). The animal use in this study was approved by the Committee of Animal Experimental Ethics of the First Affiliated Hospital of Medical College, Shihezi University, China. The living environment was as follows: 12 h of light/dark cycle, room temperature of 20–25°C, 60–70% humidity, free food and water, and a well-ventilated environment. The rats were permitted to adapt to the surrounding environment for 1 week before use. Each animal was utilized only once. All protocols were approved by the Animal Ethics Committee of the First Affiliated Hospital of Shihezi University School of Medicine (approval no. A2018-165-01) on February 26, 2018, and consistent with the Guidelines for the Care and Use of Laboratory Animals, which was published by the US National Institutes of Health.

### Intraplantar and Intrathecal Injections

The intraplantar (i.p.) injections in this study were based on a previous method (Valencia-de Ita et al., 2006). These injections were conducted to observe the protein changes in the L_4__–__6_ ganglion, because the nerves that dominate the plantar are emitted from L_4__–__6_. Under 4% sevoflurane anesthesia, the rats were injected subcutaneously with capsaicin (3 mg/ml) to simulate acute neuropathic pain. Rat thigh retraction was immediately observed, and the tip of the needle remained under the skin for a few seconds. After the drug was completely absorbed, the needle was pulled out. At this time, the rat’s thigh and foot curled up. When the rats awoke for a few seconds, follow-up experiments were performed.

Intrathecal catheterization was performed on normal rats by using the method of the author’s laboratory group (Chen et al., 2019). The rats were anesthetized and placed in a prone position, their skin was prepared, and a longitudinal incision was created above L_5__–__6_. The tissues were separated in turn, and a PE-10 catheter was used to pass through the intervertebral space. If the tail or hind limbs were twitched and a clear cerebrospinal fluid outflow was observed, the catheter was fixed and passed through the subcutaneous tunnel in the neck, at the exposed area of approximately 2 cm. When the rats awoke, 10 μl of 2% lidocaine was injected through the catheter. If lower limb paralysis occurred within 30 s after injection and recovery was observed, the placement was judged as successful (Ide et al., 2020). The rats were divided into groups after complete recovery.

### Grouping and Administration Methods

This study was divided into two parts. In the first part, was rats were randomized into the control group, capsaicin group, and bumetanide + capsaicin group. In the second part, they were randomized into the control group, capsaicin group, and U0126 + capsaicin or GF109203X + capsaicin group. Bumetanide, U0126, and GF109203X are inhibitors of NKCC1, MEK/ERK, and PKC, respectively (He et al., 2014).

The method of administering the inhibitors was as follows: capsaicin was injected subcutaneously 2 h after intrathecal injection of 20 μl of bumetanide (5 μg/μl) (He et al., 2014; Gao et al., 2019). Then, 10 μl of U0126 (1 μg/μl) was administered intrathecally for 30 min before capsaicin was administered to the plantar (Tian et al., 2020; Li Z. Y et al., 2017), while 20 μl of GF109203X (0.018 μg/μl) was administered intrathecally for 1.5 h before capsaicin was administered to the plantar (Hang et al., 2017; Zhang et al., 2004).

### Pain-Related Behavioral Test

Rats were randomly selected from the groups at 1 h before surgery and 10, 30, 60, and 120 min after surgery to detect the thermal and cold pain thresholds.

The thermal pain threshold was operated following the method of the authors’ group (Chen et al., 2019). In a quiet environment, the rats were individually placed in a transparent box to adapt to the surrounding environment for 10–15 min. Then, the skin of the capsaicin side was vertically exposed to a heat source, that is, a heat-irradiation stimulator. Recording was started when the irradiation began. The time for lifting did not exceed 30 s, and the stimulation intensity was the same when measured. The measurement was repeated three times at each time point, with at least 5 min interval, and the average was referred to as the thermal withdrawal latency (TWL) (He et al., 2019). The lower the TWL value is, the more severe the hyperalgesia of the rat under thermal stimulation.

The method used to determine the cold pain threshold was the same as the one used for the thermal pain threshold (Chen et al., 2019). The rats were placed in a prepared cage. After they were silenced, 0.1 ml of acetone was carefully dropped onto the skin of their left hind foot injected with capsaicin. The time from when the rats were lifted to the time when they placed their feet down was recorded and referred to as the cold stimulation time. The measurement was repeated three times at each time point, with at least 5 min interval, and the average was denoted as the cold withdrawal latency (CWL). The higher the CWL value is, the more severe the hyperalgesia of the rat under cold stimulation.

### Immunofluorescence

The rats were anesthetized with 4% sevoflurane. The thoracic cavity was cut to expose the heart and then perfused with 4% paraformaldehyde. The rats were observed for stiffness in the limbs, head, and neck. L_4__–__6_ DRGs were placed in 4% paraformaldehyde, fixed for 12 h, and embedded in paraffin. Subsequently, paraffin sections (sheet thickness of 5 μm) were prepared and routinely dewaxed. Antigenic repair was performed using citric acid. The sections were blocked with PBS–Triton 0.3% and 5% BSA and incubated overnight at 4°C with the following primary antibodies: mouse anti-TRPV1 (1:50, Abcam), rabbit anti-NKCC1 (1:100, Cell Signaling Technology), and rabbit anti-p-NKCC1 (1:100, Merck Millipore). The sections were then washed with PBS and incubated for 1 h at room temperature with Alexa Fluor 488- or Alexa Fluor 594-conjugated secondary antibodies (1:200; MultiSciences Biotechnology). Then, the sections were washed with PBS again. The fluorescence intensity and count were taken under a LSM 510 confocal microscope (Carl Zeiss Company, Germany). A field was randomly selected from all positive cells to calculate the average fluorescence intensity. Then, a statistical graph was drawn.

### Western Blotting

Western blotting (WB) was used to determine the total proteins from L_4__–__6_ DRGs. First, the total protein was extracted, and the protein concentration was determined in accordance with the BCA method. It was heated at 100°C for 10 min to be denatured, and the phosphorylated protein was heated at 95°C for 8 min. Protein samples (20 μl) were loaded to SDS-PAGE. After electroporation to a 0.45-nm PVDF membrane, 5% skim milk was blocked for 2 h. The following primary antibodies were incubated at 4°C overnight: mouse anti-GAPDH (1:1,000; Zsbio Commerce Store), rabbit anti-NKCC1 (1:1,000; Cell Signaling Technology), rabbit anti-p-ERK1/2 (1:1,000; Cell Signaling Technology), and rabbit anti-p-NKCC1 (1:1,000; Merck Millipore). Horseradish peroxidase-coupled secondary antibody (1:20,000; Zsbio Commerce Store) was incubated for 2 h at room temperature and then washed with TBST. Subsequently, a freshly prepared ECL chemiluminescence reagent was added on the Fluorchem 9900 imaging system. ImageJ software was used to analyze the results.

### PCR

Total RNA was extracted from the L_4__–__6_ DRGs of rats by using TRIzol reagent (Invitrogen) in accordance with the protocols from the manufacturer and reverse transcribed into cDNA by using a qRT-PCR kit (Invitrogen) at 42°C for 60 min and 70°C for 5 min. The following primers were used for qPCR amplification: β-actin, forward 5′-CGTAAAGACCTCTATGCCAACAG-3′, reverse 5′-AGCCACCATCCACACAGAG-3′; and NKCC1, forward 5′-CAGTGGTGGTTCTTCTGGGC-3′ and reverse 5′-GGGCTTCTTGCTGTCCAGTG-3′. The thermocycling conditions consisted of 3 min of hot-start enzyme activation at 95°C, followed by 40 cycles of PCR at 95°C for 30 s (denaturation) and 58°C for 20 s (annealing) by using SYBR Green Real-Time PCR Master Mix (Toyobo Co., Ltd., Osaka, Japan). Amplification was confirmed by the presence of a single peak in the melting temperature analysis and linear amplification throughout the PCR cycles. The 2^–(ΔΔ*t)*^ method was used to analyze the relative mRNA expression of the target genes. The mRNA level of β-actin was used as the internal control.

### Acute Isolation of DRG Neurons

The method was adopted from the authors’ laboratory (Tan et al., 2020). After the anesthetized rats were sacrificed, the L_4__–__6_ segment spine was taken and placed in extracellular fluid saturated with oxygen (150 mmol/l NaCl, 5 mmol/l KCl, 1 mmol/l MgCl_2_, 10 mmol/l HEPES, 10 mmol/l D-glucose, 20 mmol/l sucrose, and 3.32 mmol/l CaCl_2_; 7.2–7.4 pH; osmotic pressure of 320–350 mOsm). Fine eye scissors and hairspring forceps were used to separate, trim, and cut the DRG sufficiently. Then, digestive enzymes (0.24 mg/ml of type III trypsin and 0.6 mg/ml of type IA collagenase dissolved in DMEM) were used in a 37°C water bath and digested for 3–4 min. Afterward, a small amount of trypsin inhibitor was added to terminate the digestion immediately. The cells were left to adhere to the wall. Then, an oxygen-saturated extracellular fluid was added after the cell wall was fully attached.

### Patch Clamp

Dorsal root ganglion cells with small and medium diameters, smooth membrane surfaces, and good translucency were selected for the experiments. Coverslips with DRG neurons were installed in a small flow-through chamber positioned on the stage of an inverted microscope (Nikon Eclipse Ti, Tokyo, Japan). The coverslips were continuously infused with a gravity-driven bath solution. A glass pipette was prepared using a Sutter p-87 puller (3–5 m). The resistance of this glass pipette was approximately 3–5 MΩ. Currents were recorded using a Multi Clamp700B amplifier (Axon Instruments, United States), filtered at 10 kHz with a low-pass filter, and digitized with Digidata 1550A (Axon Instruments, United States). pCLAMP 10.5 (Axon Instruments) was used for data acquisition. The drug delivery system was conducted by moving the discharge tube of the rapid liquid exchange device through the micromanipulator. The diameter of each nozzle of the discharge tube was 0.5 mm. Under the whole-cell mode, the drain tube containing GABA was opened and quickly moved to the same plane of the detected cells. The nozzle distance to the recorded cells was approximately 100 m. The inverted microscope showed that the perfusion liquid completely covered the test cells. After 5 s of GABA perfusion, the nozzle was removed and the dosing tube was closed. The activation current of GABA_A_ receptor was then recorded, and the extracellular fluid tube mouth in the open state was moved to the detection cell elution for 5 min. The sodium chloride bath solution contained 150 mM of NaCl, 5 mM of KCl, 1 mM of MgCl_2_, 2.5 mM of CaCl_2_, 10 mM of glucose, 20 mM of sucrose, and 10 mM of HEPES (pH 7.40, with NaOH). The potassium chloride pipette solution contained 130 mM of KCl, 10 mM of NaCl, 1.2 mM of MgCl_2_, 2 mM of CaCl_2_, 7.5 mM of glucose, 5 mM of EGTA, and 10 mM of HEPES (pH 7.30, with KOH). Whole cells were formed after breaking the plasma membrane under the pipette tips. Experiments were conducted at room temperature between 22 and 30°C.

### Chloride Ion Fluorescent Probe

L_4__–__6_ DRG specimens were collected from different groups. In accordance with the experimental procedure of Batti et al. (2013) and the improvement methods from the authors’ laboratory (Tan et al., 2020), the animals were sacrificed using 4% sevoflurane, with immediate stripping of L_4__–__6_ DRG. Then, samples were cut with eye scissors, subjected to enzyme digestion, and centrifuged. After the clear liquid was removed, the specimens were placed on a 12-well plate that had been precoated with poly-D-lysine (Beyotime) for fixing cells. Then, N-ethoxycarbonylmethyl-6-methoxyquinolinium bromide (MQAE) buffer was added and incubated at 37°C for 30 min, and the specimens were cleaned with a buffer. A fluorescence microscope was used to record the fluorescence intensity. When the concentration of Cl^–^ in the cell increased, the fluorescence intensity of MQAE was proportionally reduced.

### Statistical Analysis

Experimental data were presented as the mean ± standard deviation. SPSS 23.0 software (SPSS Inc., Chicago, IL, United States) was used for all statistical analyses, and the diagram of the experimental data was created on GraphPad Prism 7.0 software. Regarding the western blot, PCR, and patch clamp data, two-group comparisons were conducted using independent-sample *t*-tests; comparison of three or more groups was performed using one-way ANOVA; and repeated measures ANOVA was used to compare the behavior of pain (TWL and CWL) between groups. *P* < 0.05 was considered as statistically significant.

## Results

### NKCC1 Transporter Protein Participates in Inflammatory Hyperalgesia Induced by Plantar Injection of Capsaicin in Rats

**FIGURE 1:**
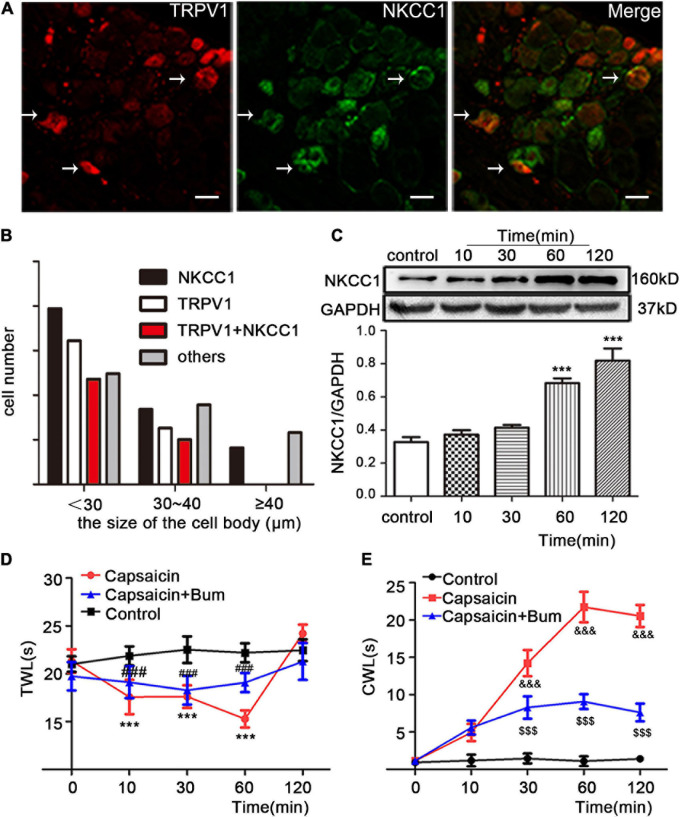
The effect of NKCC1 transporter on capsaicin-induced inflammatory pain. **(A)** Immunofluorescence (×40) showed the expression distribution of TRPV1 and NKCC1 transporter in the control group. The arrow shows the cells co-expressed by two proteins. Scale bar = 25 μm. **(B)** Cell size-dependent distribution of rat DRG neurons expressing TRPV1 and NKCC1, TRPV1, or NKCC1 (*n* = 817 cells). **(C)** Effect of capsaicin on the expression of NKCC1 transporter protein in DRG neurons at different time points in rats (***P < 0.001 compared with the control group). **(D,E)** Pain behavioral changes after plantar injection of capsaicin: **(D)** TWL and **(E)** CWL. *t* = 12.628, ***P < 0.001 and *t* = –23.59, ^&⁣&⁣&^*P* < 0.001 compared with the control group; *t* = –6.918, ^###^*P* < 0.001 and *t* = 15.32, ^$$$^*P* < 0.001 compared with the capsaicin group (*n* = 6). The data is normally distributed. TWL, thermal withdrawal latency; CWL, cold withdrawal latency; NKCC1, sodium and potassium chloride co-transporter; TRPV1, transient receptor potential vanilloid 1; DRG, dorsal root ganglion; con, control; Bum, bumetanide.

Dorsal root ganglions were divided on the basis of cell diameter: small cells (<30 μm), medium cells (30 μm ≤ diameter < 40 μm), large cells (≥40 μm). First, the distribution of TRPV1 (red) and NKCC1 transporter (green) in normal DRG neurons and the co-expression of TRPV1 and NKCC1 transporter in the same DRG neuron were detected (Figure 1A). The results showed that TRPV1 and NKCC1 transporters were not only expressed in DRG neurons but also co-expressed in the same DRG neuron. Among them, 27.4% (224/817) of DRG neurons were TRPV1 immunoreactive, 39.4% (322/817) of DRG neurons were NKCC1 transporter immunoreactive, and 20.6% (168/817) of DRG neurons were not only TRPV1 immunoreactive but also NKCC1 transporter immunoreactive (Figure 1B), indicating that NKCC1 transporter and TRPV1 were co-expressed. Previous reports have shown that TRPV1 and mRNA of NKCC1 transporters are co-expressed (Price et al., 2006). Figure 1B also shows the immunoreactivity of TRPV1 and NKCC1 transporters in DRG neurons with different diameters. Subsequently, the pain behavior of the model rats was tested. After capsaicin was injected in the plantar, the TWL of the rats decreased and the CWL was prolonged, indicating sensitivity to pain. After bumetanide was intrathecally administered, TWL (Figure 1D) and CWL (Figure 1E) were relieved from the feeling of pain. Thermal hyperalgesia appeared significantly altered at 60 min time (22.20 ± 0.99 in the control group vs. 15.28 ± 0.90 in the capsaicin group, *P* = 0.000, *n* = 6; 15.28 ± 0.90 in the capsaicin group vs. 19.07 ± 1.00 in the bumetanide + capsaicin group, *P* = 0.000, *n* = 6). Cold hyperalgesia appeared significantly altered at 60 min time (1.08 ± 0.66 in the control group vs. 21.73 ± 2.04 in the capsaicin group, *P* = 0.000, *n* = 6; 21.73 ± 2.04 in the capsaicin group vs. 9.01 ± 1.11 in the bumetanide + capsaicin group, *P* = 0.000, *n* = 6). WB results showed that the expression of NKCC1 transporter protein increased after plantar injection of capsaicin. The expression of NKCC1 transporter protein was significantly higher at 60 and 120 min in the control group (0.32 ± 0.03 in the control group vs. 0.68 ± 0.03 at 60 min, *P* = 0.000, *n* = 6; 0.81 ± 0.07 in the control group vs. 0.32 ± 0.03 at 120 min, *P* = 0.000, *n* = 6) (Figure 1C). The changes of NKCC1 transporter protein expression were consistent with the behavioral results. These results suggest that NKCC1 transporter is involved in the pain response induced by capsaicin-activated TRPV1.

### Change in Distribution and Protein Expression of DRG NKCC1 and p-NKCC1 Transporter Proteins After Capsaicin Injection

**FIGURE 2:**
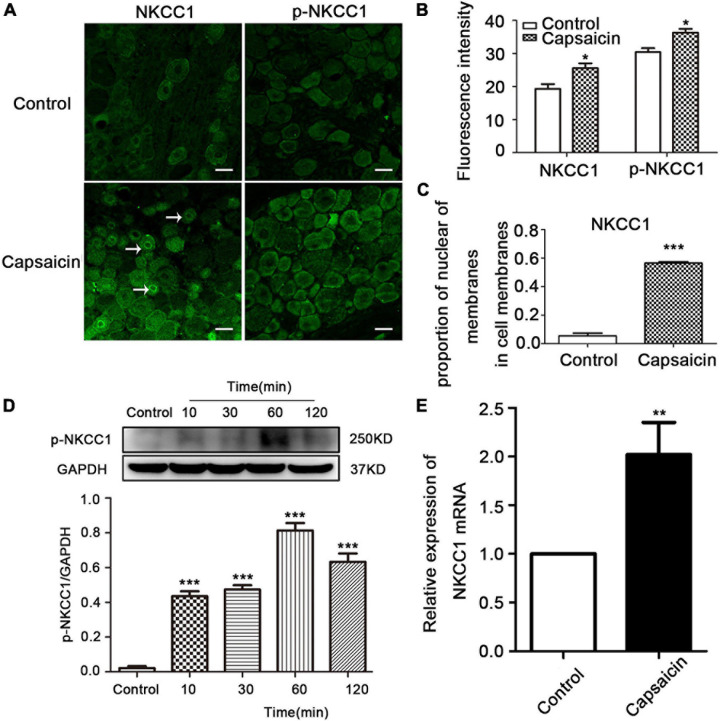
The effect of capsaicin on the expression and distribution of NKCC1 and p-NKCC1 transporter proteins in DRG neurons. **(A)** Immunofluorescence showed the expression levels of NKCC1 and p-NKCC1 after capsaicin administration. Scale bar = 25 μm. **(B)** Fluorescence intensities of NKCC1 and p-NKCC1. **(C)** Proportion of nuclear membrane expression on the cell membrane of NKCC1 in the control and capsaicin groups. **(D)** The effect of plantar injection of capsaicin on the expression of p-NKCC1 in rats at different time points. **(E)** The expression of NKCC1 transporter proteins at the mRNA level after capsaicin administration *P < 0.05, **P < 0.01, and ***P < 0.001 compared with the control group (*n* = 6). The data is normally distributed. NKCC1, sodium and potassium chloride co-transporter; TRPV1, transient receptor potential vanilloid 1; DRG, dorsal root ganglion.

The expression and distribution of NKCC1 and p-NKCC1 transporter were detected by WB, immunofluorescence, and PCR. Immunofluorescence results showed that the immunofluorescence intensity of NKCC1 transporter protein expression in the capsaicin group was higher than that in the control group (19.33 ± 1.41 in the control group vs. 25.57 ± 1.46 in capsaicin group, *P* = 0.049, *n* = 6). Compared with the control group, the expression of p-NKCC1 transporter protein in the cell membrane and cytoplasm of small and medium cells was increased (30.43 ± 1.19 in the control group vs. 36.33 ± 1.12 in the capsaicin group, *P* = 0.037) (Figures 2A,B). It is worth noting that the expression of NKCC1 transporter protein on the nuclear membrane of small and medium-sized DRG cells was significantly increased compared with the control group (0.05 ± 0.02 in the control group vs. 0.56 ± 0.01 in the capsaicin group, *P* = 0.000, *n* = 6) (Figures 2A,C). WB results showed that compared with the control group, the expression of p-NKCC1 transporter protein in the capsaicin group was increased, and the highest expression was at 60 min (Figure 2D, 0.02 ± 0.01 in the control group vs. 0.81 ± 0.04 in the 60-min group, *P* = 0.000, *n* = 6; 0.02 ± 0.01 in the control group vs. 0.63 ± 0.05 in the 120-min group, *P* = 0.000, *n* = 6). This result was basically consistent with the change of NKCC1 transporter protein (Figure 1C). PCR results showed that the mRNA level of NKCC1 transporter protein in the capsaicin group was significantly higher than that in the control group (1 in the control group vs. 2.05 ± 0.55 in the capsaicin group, *P* = 0.006, *n* = 6) (Figure 2E). These results suggest that capsaicin-activated TRPV1 can increase the expression of NKCC1 transporter protein at mRNA and protein levels and increase the phosphorylation level of NKCC1 transporter protein in DRG neurons.

### Effect of Capsaicin on the Function of NKCC1 Transporter in Rat DRG Neurons

**FIGURE 3:**
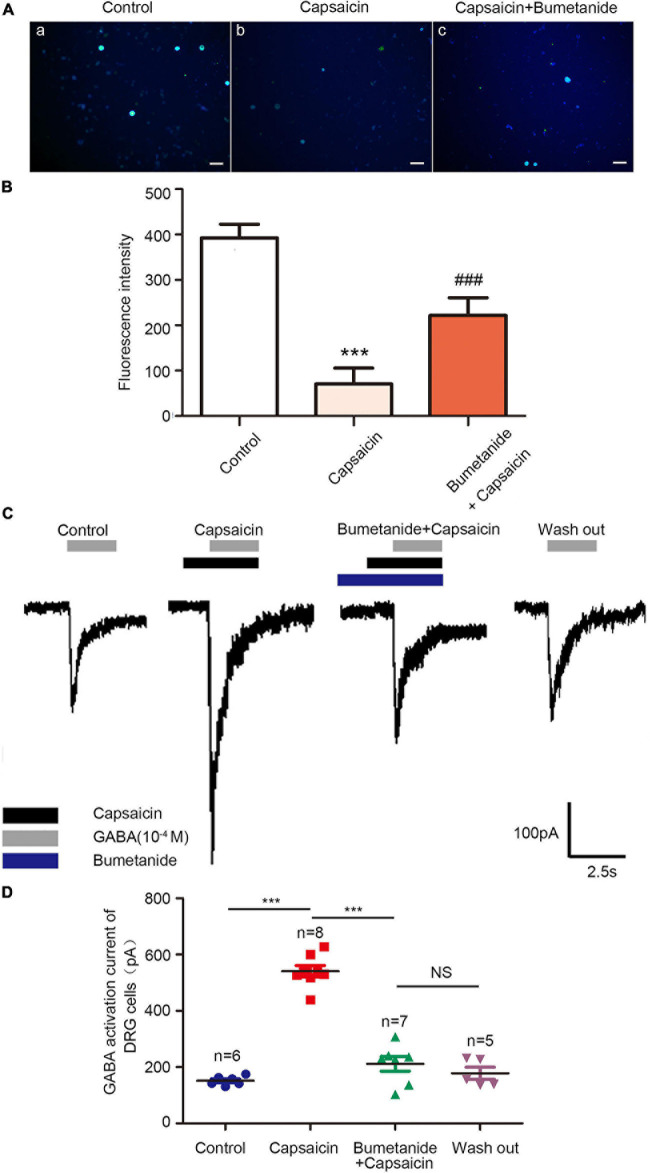
Chloride ion fluorescence probe and GABA-activated current in DRG neurons of the control group, capsaicin group, and bumetanide + capsaicin group. Chloride ion fluorescent probe showed **(A)** the changes in the intracellular chloride ion concentration in the DRG of the control group (a), capsaicin group (b), and bumetanide + capsaicin group (c). Scale bar = 100 μm. **(B)** Statistical diagram of fluorescence intensity of each group (*n* = 3) (*F* = 21.51, ***P < 0.001 compared with the control group, and^ ###^*P* < 0.001 compared with the capsaicin group). **(C)** GABA currents recorded using patch clamps in the control group, capsaicin group, bumetanide + capsaicin group, and washout group. **(D)** Scatter plots of GABA-activated current of DRG cells in different groups. *F* = 86.77, ***P < 0.01 compared with the capsaicin group (*n* = 5–8). The data is normally distributed. DRG, dorsal root ganglion.

Considering NKCC1 is an electrically neutral sodium, potassium, and chlorine ion co-transporters, dynamic changes in ions play a key role in their functions. Therefore, the mechanism of NKCC1 was explored, combined with previous literature methods. GABA-activated current indirectly reflects the change of Cl^–^ concentration in DRG cells. The higher the chloride concentration in neurons, the greater the inward current (depolarization) produced by GABA_A_ receptor activation. In the experiment, L_4__–__6_ DRG neurons of normal rats were selected, and 20–50-μm diameter DRG neurons were selected under a microscope. Under voltage clamp, GABA and capsaicin were administered through the dosing tube for 2.5 s (10 μm) to detect GABA activation current. Compared with the control group, the GABA activation current increased significantly after capsaicin pretreatment. If bumetanide (10 μm) was incubated for 15 min and then preperfused with capsaicin for 2.5 s, the GABA activation current was significantly reduced compared with that of capsaicin only. As shown in Figures 3C,D, the values of GABA-activated current in each group were as follows: control group (151.93 ± 16.49 pA), capsaicin group (540.77 ± 56.28 pA), bumetanide + capsaicin group (211.43 ± 24.36 pA), and washout group (178.10 ± 47.72 pA) (among them, the control group vs. capsaicin group, *P* = 0.000; capsaicin group vs. bumetanide + capsaicin group, *P* = 0.000, *n* = 5–8). These results indicate that the GABA-activated current increases after capsaicin pretreatment, which indicates the increase of intracellular chloride in DRG neurons.

In addition, the changes in chloride ion concentration in DRG neurons were determined using a chloride ion fluorescence probe. The results showed that the concentration of chloride ion in the DRG neurons of the capsaicin group was significantly higher than that of the control group; however, the chloride concentration in DRG neurons was decreased in the bumetanide + capsaicin group. Among them, 392.43 ± 52.31 in the control group vs. 70.80 ± 60.05 in the capsaicin group, *P* = 0.000, 70.80 ± 60.05 in the capsaicin group vs. 221.92 ± 67.03 in the bumetanide + capsaicin group, *P* = 0.000, *n* = 8 (Figures 3A,B). These results suggest that capsaicin increases the chloride concentration in DRG neurons by enhancing the chloride transport function of NKCC1 transporter.

### Capsaicin Increases the Expression and Phosphorylation of NKCC1 Transporter Protein Through PKC/ERK Pathway

**FIGURE 4:**
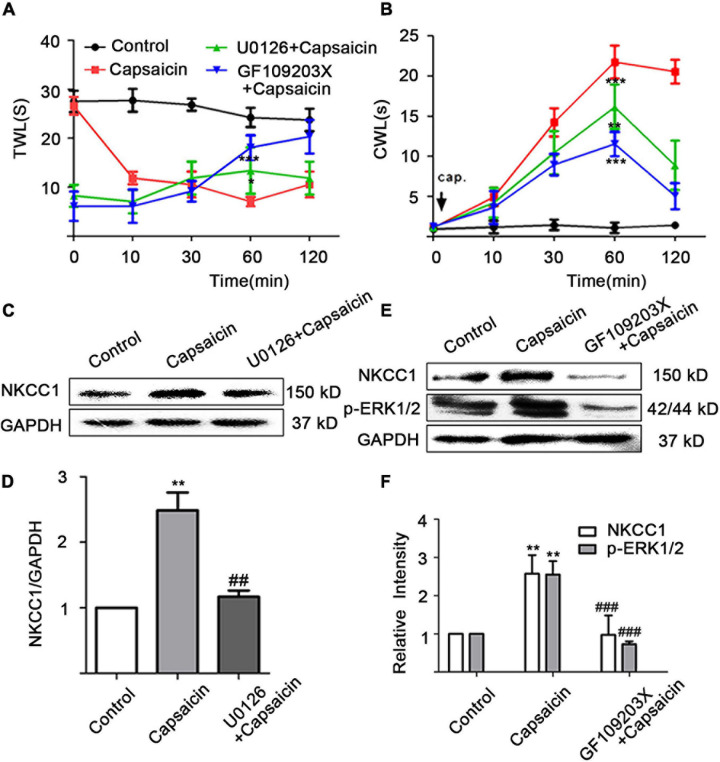
Capsaicin increases the expression of NKCC1 transporter protein through the PKC/ERK pathway. Intrathecal injection of U0126 for 1 h or intrathecal injection of for 2 h reduced **(A)** thermal and **(B)** cold hyperalgesia induced by the administration of capsaicin from the soles of the paw of rats (*n* = 6). **(A)** *t* = –2.745, *P < 0.05, U0126 + capsaicin vs. capsaicin; *t* = –7.55, ***P < 0.001, GF109203X + capsaicin vs. capsaicin. **(B)** *t* = 3.965, **P < 0.01, U0126 + capsaicin vs. capsaicin; *t* = 9.86, ***P < 0.001, GF109203X + capsaicin vs. capsaicin. **(C,D)** The effect of MEK blocker (U0126) on NKCC1 transporter protein expression. *F* = 23.86, **P < 0.01, compared with the control group, and ^##^*P* < 0.01, compared with the capsaicin group. **(E,F)** The effect of the expression levels of NKCC1 transporter protein and p-ERK after PKC blocker (GF109203X) was administered. *F*(p-ERK) = 20.24, *F*(NKCC1) = 10.25, **P < 0.01 compared with the control group and ^###^*P* < 0.001 compared with the capsaicin group. The data is normally distributed. TWL, thermal withdrawal latency; CWL, cold withdrawal latency; NKCC1, sodium and potassium chloride co-transporter; TRPV1, transient receptor potential vanilloid 1.

Behavioral testing showed that the administration of the MEK/ERK inhibitor (U0126) and the PKC inhibitor (GF109203X) can reverse the thermal hyperalgesia and cold hyperalgesia caused by capsaicin administration on the paw. Thermal hyperalgesia appeared significantly altered at 60 min (7.02 ± 2.12 in the capsaicin group vs. 13.38 ± 4.73 in the U0126 + capsaicin group at 60 min, *P* = 0.036; 7.02 ± 2.12 in the capsaicin group vs. 18.08 ± 2.50 in the GF109203X + capsaicin group at 60 min, *P* = 0.000) (Figure 4A). Figure 4B shows that the CWL of the capsaicin group was significantly longer than that of the control group, but the shortened CWL was reversed after pretreatment with U0126 and GF109203X. When capsaicin was used for 60 min, the CWL of the capsaicin group (21.73 ± 2.04 s, *n* = 6) was significantly longer than that of control group (2.61 ± 0.43 s, *P* = 0.000, *n* = 6), but that of the U0126 + capsaicin group (16.17 ± 2.77, *P* = 0.003, *n* = 6) and the GF109203X + capsaicin group (11.52 ± 1.51, *P* = 0.000, *n* = 6) was significantly shorter than that of the capsaicin group (Figure 4B).

A quantitative analysis of NKCC1 transporter protein showed that the expression of NKCC1 transporter protein in the capsaicin group was significantly higher than that in the control group (1 in the control group vs. 2.48 ± 0.47 in the capsaicin group, *P* = 0.002, *n* = 6), but the expression of NKCC1 transporter protein in the U0126 + capsaicin group was significantly lower than that in the capsaicin group (2.48 ± 0.47 in the capsaicin group vs.1.17 ± 0.17 in the U0126 + capsaicin group, *P* = 0.004, *n* = 6) (Figures 4C,D). After intrathecal injection of GF109203X, the significant increase of NKCC1 transporter protein and p-ERK protein expression mediated by capsaicin was reversed. The expression of NKCC1 transporter protein in the capsaicin group was significantly higher than that in the control group (1 in the control group vs. 2.58 ± 0.34 in the capsaicin group, *P* = 0.009, *n* = 6), but the expression of NKCC1 transporter protein in the GF109203X + capsaicin group was significantly lower than that in the capsaicin group (2.58 ± 0.34 in the capsaicin group vs. 0.87 ± 0.40 in the GF109203X + capsaicin group, *P* = 0.000, *n* = 6). The expression of p-ERK1/2 protein in the capsaicin group was also significantly higher than that in the control group (1 in the control group vs. 2.39 ± 0.25 in the capsaicin group, *P* = 0.008, *n* = 6), but the expression of p-ERK1/2 protein in the GF109203X + capsaicin group was significantly lower than that in the capsaicin group (2.39 ± 0.25 in the capsaicin group, 0.63 ± 0.11 in the GF109203X + capsaicin group, *P* = 0.000, *n* = 6) (Figures 4E,F). However, the total protein expression of ERK1/2 did not change (not shown). The results showed that NKCC1 transporter was the downstream protein of PKC and p-ERK.

**FIGURE 5:**
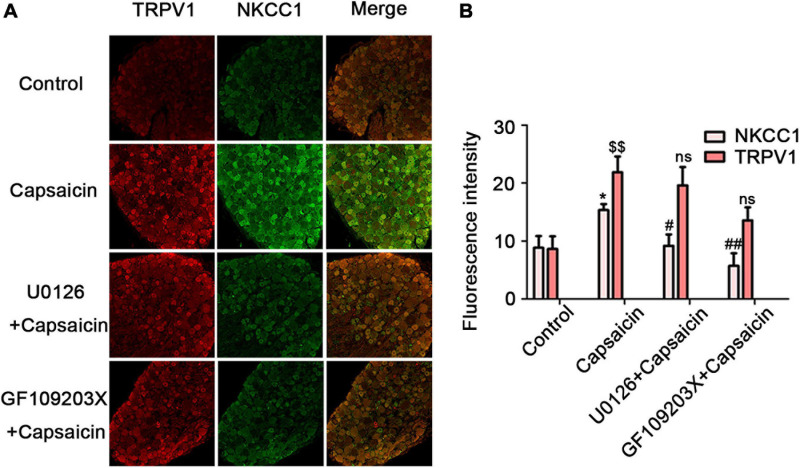
U0126 or GF109203X reverses capsaicin-induced increase in NKCC1 transporter protein expression. **(A)** Immunofluorescence showed the expression of NKCC1 transporter protein and TRPV1 receptor protein in DRG neurons. Scale bar = 50 μm. **(B)** The fluorescence optical intensity of TRPV1 protein and NKCC1 transporter protein. *F*(TRPV1) = 15.85, ^$$^*P* < 0.01, *n* = 6 and *F*(NKCC1) = 14.23, *P < 0.01, *n* = 6 compared with the control group; U0126 + capsaicin group (^#^*P* < 0.05, *n* = 6) and GF109203X + capsaicin group (^##^*P* < 0.01, *n* = 6) compared with the capsaicin group. The data is normally distributed. NKCC1, sodium and potassium chloride co-transporter; TRPV1, transient receptor potential vanilloid 1.

The immunofluorescent staining optical intensity of rat ipsilateral L_4__–__6_ DRGs of different groups was parallel to the WB expression of NKCC1 transporter protein. The fluorescence optical intensity of NKCC1 was 8.84 ± 2.03 in the control group, 15.34 ± 1.04 in the capsaicin group (compared with the control group, *P* = 0.016, *n* = 6), 9.20 ± 1.94 in the U0126 + capsaicin group (compared with the capsaicin group, *P* = 0.022, *n* = 6), 5.70 ± 2.18 in the GF109203X + capsaicin group (compared with the capsaicin group, *P* = 0.001, *n* = 6). Figure 5 also shows the optical intensity of TRPV1 in different groups (8.63 ± 2.17 in the control group vs. 21.87 ± 2.73 in the capsaicin group, *P* = 0.002, *n* = 6; 21.87 ± 2.73 in the capsaicin group vs. 19.61 ± 3.16 in the U0126 + capsaicin group, *P* = 1.000, *n* = 6, no significance; and 21.87 ± 2.73 in the capsaicin group vs. 13.57 ± 2.24 in the GF109203X + capsaicin group, *P* = 0.131, *n* = 6, no significance). It can also be seen from Figure 5 that the immunofluorescence co-expression of NKCC transporter protein and TRPV1 protein in the capsaicin group was the brightest and the most intense (Figure 5).

### Capsaicin Enhances the Function of NKCC1 Transporter Through the PKC/ERK Pathway

**FIGURE 6:**
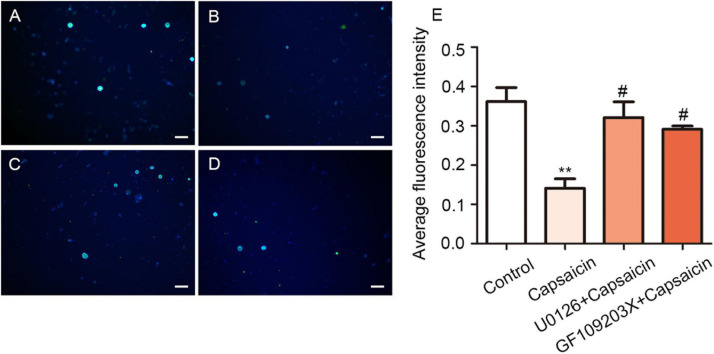
U0126 and GF109203X reverse capsaicin-induced enhancement of NKCC1 transporter function. Chloride ion fluorescent probes showed the concentration of intracellular chloride ion in rat DRG in the **(A)** control group, **(B)** capsaicin group, **(C)** U0126 + capsaicin group, and **(D)** GF109203X + capsaicin group. The fluorescence intensity was inversely proportional to the concentration of chloride ion in neurons. Scale bar = 100 μm. **(E)** Statistical graph of mean fluorescence intensity (*F* = 10.40, **P < 0.01 compared with the control group, ^#^*P* < 0.05, compared with the capsaicin group, *n* = 6). The data is normally distributed.

At the functional level, the intracellular chloride concentration was detected by chloride fluorescent probe technology to verify that capsaicin phosphorylates NKCC1 transporter through the PKC/ERK pathway and activates its function (Tan et al., 2020). Meanwhile, the fluorescence intensity of chloride ion decreases proportionally according to the increase of intracellular chloride ion concentration. The fluorescence intensity of the capsaicin group was significantly lower than that of the control group (0.36 ± 0.06 in the control group vs. 0.14 ± 0.04 in the capsaicin group, *P* = 0.005, *n* = 6). The fluorescence intensity of the U0126 + capsaicin and GF109203X + capsaicin groups was significantly higher than that of the capsaicin group (0.14 ± 0.04 in the capsaicin group vs. 0.32 ± 0.06 in the U0126 + capsaicin group, *P* = 0.016, *n* = 6; 0.14 ± 0.04 in the capsaicin group vs. 0.29 ± 0.01 in the GF109203X + capsaicin group, *P* = 0.045, *n* = 6) (Figure 6). This result indicates that after capsaicin administration, TRPV1 was activated, which enhanced the function of NKCC1 transporter and increased the intracellular chloride concentration. After pretreatment with ERK/MEK inhibitor (U0126) and PKC inhibitor (GF109203X), the intracellular chloride fluorescence intensity increased compared with the capsaicin group, indicating that the increase of intracellular chloride concentration was reversed (Figure 6E).

## Discussion

The findings of this study were as follows: (1) pain behavior studies showed that compared with the control group, TWL decreased and CWL prolonged in the capsaicin group, but the effect of capsaicin was partially reversed after pretreatment with bumetanide, U0126, or GF109203X; (2) immunofluorescence and WB showed TRPV1 and NKCC1 were co-expressed in the pain-related small and medium DRG neurons of control group rats; (3) after TRPV1 was activated, the expression of NKCC1 on the nuclear membrane increased, while that of p-NKCC1 in the cytoplasm or in the membrane increased significantly; (4) patch clamp results showed that the GABA activation current increased significantly compared with that of control rats, and the effect of capsaicin was partially reversed after pretreatment with bumetanide; (5) fluorescent probe results showed that the Cl^–^ concentration in the DRG neurons of the capsaicin group increased significantly, but the effect of capsaicin was partially reversed after pretreatment with bumetanide, U0126, or GF109203X; and (6) TRPV1 activation by capsaicin may up-regulate the expression and the functional activity of NKCC1 (p-NKCC1) through the PKC (p-ERK) signal transduction pathway. These results suggested that the activation of TRPV1 can cause changes in NKCC1 and aggravate pain in experimental animals.

Many studies have shown that TRPV1 is involved in various pain models related to transient receptor potential family (Patapoutian et al., 2009; Leo et al., 2017). Intrathecal injection of AVV9 shRNA TRPV1 could reduce hyperalgesia induced by a peripheral nerve injury model in mice (Hirai et al., 2014). The MZF1 in the dorsal root ganglia promotes the development and maintenance of neuropathic pain by regulating TRPV1 (Xing et al., 2019). After the periphery nerve is activated, a large amount of calcium and sodium ions flow in the neuron from extracellular, and the intracellular potassium ion outflow caused by the potassium channel opening is inhibited (Wu et al., 2013). The influx of calcium ions has become an important condition for triggering spontaneous discharge of cells (Li et al., 2007; Bavassano et al., 2013; Mathie and Veale, 2015). This phenomenon may be one of the reasons why TRPV1 activation produces pain. Also, an inflammatory response occurs around the stimulation site, and primary sensory neuron ends release neuropeptides such as P (SP), causing neurogenic inflammatory responses (Kilo et al., 1997; Derow et al., 2007). Then, P (SP) substances could bind with NK-1 to regulate TRPV1 and play a positive role (Zhang et al., 2007). This phenomenon may be the other one of the reasons why TRPV1 activation produces pain. However, a conclusive evidence that TRPV1 directly promotes pain does not exist, but the up-regulation of TRPV1 contributes to the development of pain and maintenance (Dai, 2016). These findings suggest that there may be other regulatory mechanisms involved in TRPV1-mediated pain, which needs further study.

Early studies have shown that DRG neurons can be divided into three categories according to the size of the cell body. Typically large neurons emit coarse myeloid fibers (Aα/Aβ, diameter ≥ 40 μm) and fast conduction velocities, which are related to proprioception; middle neurons emit fine myelin fibers (Aδ, 30 μm ≤ diameter < 40 μm), which are related to touch; small neurons mainly emit nociceptive unmyelinated fibers (C fiber, diameter < 30 μm), which are related to sensation of temperature and pain (Fang et al., 2005; Paddock et al., 2018; Madden et al., 2020). Different types of DRG neurons (with different cell diameters) not only transmit different proprioception but also have different electrophysiological characteristics (such as action potential shape, etc.), bioactive substances synthesized and secreted by neurons (such as neurotransmitters, etc.), and specific protein and membrane receptor types expressed by neurons (such as special markers, etc.) (Fang et al., 2005; Paddock et al., 2018; Madden et al., 2020). Previous studies have shown that NKCC1 mRNA and TRPV1 are co-expressed on the small and medium DRG neurons of a normal rat (Price et al., 2006). The rapid activation of NKCC1 and the transport of NKCC1 may be the basis for the initial development and subsequent maintenance of hyperalgesia caused by capsaicin (Galan and Cervero, 2005). In the present study, the sensitivity to cold and heat pain was enhanced after the administration of capsaicin to the plantar, and the administration of bumetanide in the sheath could significantly inhibit the hyperalgesia caused by capsaicin and after activating TRPV1. In the capsaicin group, the expression of NKCC1 and p-NKCC1 protein in DRG increased, but the expression of NKCC1 protein increased significantly around the cell nuclear membrane, while the expression of p-NKCC1 in the cytoplasm and cell membrane increased significantly, which may be related to the activation of TRPV1 by exogenous activators (such as capsaicin) involved in the process of pain generation, while maintaining the transcription, translation, and post-translational modification of NKCC1 transporter. This result was also reflected in the PCR results of NKCC1, which eventually led to TRPV1-stimulated NKCC1 protein synthesis. The results showed that NKCC1 protein was rapidly transported from the nuclear membrane to the cytoplasm or cell membrane after phosphorylation. These results suggest that TRPV1 activation can increase the expression of NKCC1 mRNA and protein, suggesting that this activation may play an important role in the maintenance of pain.

**FIGURE 7:**
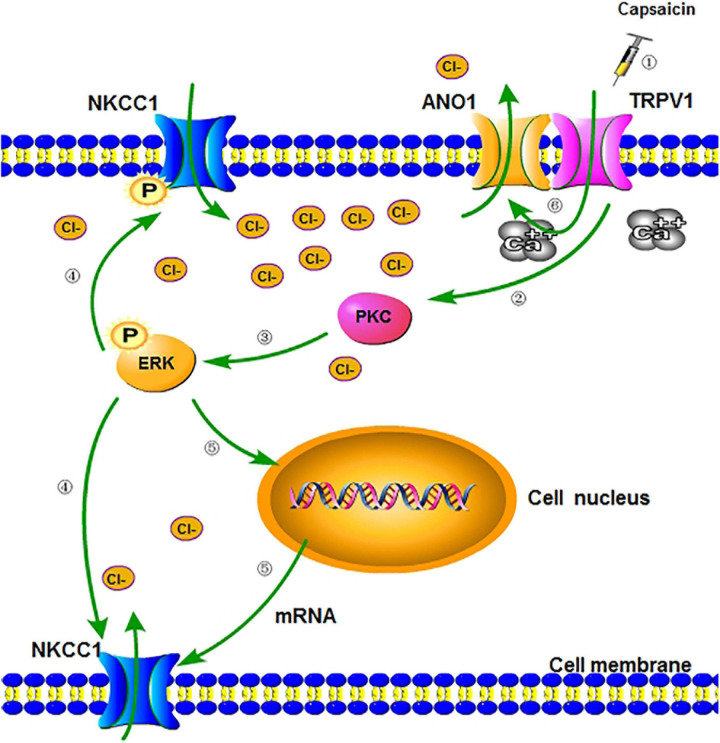
A schematic diagram of regulation of NKCC1 expression and function after activation of TRPV1. (1) Peripheral administration of capsaicin-activated TRPV1. (2) When TRPV1 is activated, the channel opens, allowing a large number of cations to flow into cells (including calcium ion) (Takayama et al., 2015) and indirectly activating PKC through signal transduction. (3) PKC activates and phosphorylates downstream ERK, which is involved in receptor-regulated ERK pathways and the activation of transcription factors. (4) p-ERK partially phosphorylates the downstream NKCC1 transporter and enhances the ion transport function of the transporter (including chloride transport to cells). (5) Part of p-ERK can enter the nucleus, increase the transcription of NKCC1 mRNA, and translate more NKCC1 transporter proteins. (6) The activation of TRPV1 causes intracellular calcium influx, which can directly increase the opening of calcium-activated chloride channel (ANO1) bound to TRPV1 (Takayama et al., 2015), make a large amount of chloride outflow, and cause more severe depolarization of neurons (inflammatory hyperalgesia). NKCC1, sodium chloride and potassium chloride cotransporter; TRPV1, transient receptor potential vanillin 1; ANO1, anoctamin-1; PKC, protein kinase C; ERK, extracellular regulated protein kinase.

The electrophysiological results of this study showed that the GABA activation current of the DRG neurons in the capsaicin group significantly increased. The results of the chloride ion probe also showed that the chloride ion concentration in DRG neurons of the capsaicin group significantly increased, but pretreatment with bumetanide can reverse all the effect of capsaicin on TRPV1. This finding suggested that after capsaicin activates TRPV1, NKCC1 increases its expression and the ability to transport Cl^–^ is significantly enhanced, which keeps DRG neurons at a higher level of Cl^–^ concentration. Finally, bumetanide administration could reduce the amount of GABA activation current increasing induced by capsaicin, and the concentration of chloride ion in DRG neurons was correspondingly reduced. Two reasons may be speculated as to how an increase in Cl^–^ concentration in DRG neurons caused pain intensification after activating TRPV1: first, at the peripheral end of the primary sensory afferent, due to the interactions of TRPV1 and ANO1 (ANO1 protein is one of the molecular bases for calcium-activated chlorine channels) (Tominaga and Takayama, 2014; Takayama et al., 2015), TRPV1 could trigger the opening of ANO1 when the Cl^–^ concentration in the DRG neurons increases (Figure 7); in addition, the positive feedback effect of Cl^–^ efflux-mediated depolarization is strengthened, thereby making the peripheral nociceptors more prone to burst action potentials (APs) (Takayama et al., 2015; Palhares et al., 2017; Takayama and Tominaga, 2018). Second, at the central terminal of the primary sensory afferent, the primary afferent excitement activates the intermediate inhibitory neurons in the dorsal horn of the spinal cord, and GABA is released in the end. As intermediate inhibitory neurons, GABA neurons normally participate in regulating primary sensory transmission. They also reduce the membrane potential of the presynaptic membrane at the central terminal of the primary sensory nerve ending through the synapses formed by the GABA nerve fibers and the central terminal of the primary sensory terminal, resulting in primary afferent depolarization, which reduces the Ca^2+^ influx of the presynaptic membrane. The excitatory neurotransmitters that drive synaptosomes to fuse into synaptic vesicles are reduced, and presynaptic inhibition and pain-inhibiting effects occur. However, when TRPV1 is activated, the Cl^–^ concentration in the DRG neurons considerably increases due to the increase in NKCC1 activity. The GABA released by intermediate inhibitory neurons activates GABA_A_ receptors, opens Cl^–^ channels, and increases Cl^–^ efflux (inward current increase), as shown in Figures 3C,D. Thus, the membrane potential of the presynaptic membrane at the central end of the primary sensory end undergoes a sharp, excessive depolarization and reaches the threshold potential to trigger the AP, which could cause DRR pain sensitivity (Tan et al., 2020).

Earlier studies have also confirmed that PKC is involved in receptor-regulated ERK pathways and the activation of transcription factors (Liu et al., 2007; Seybold, 2009; Wang et al., 2012; Cornelison et al., 2016). The PKCγ-ERK1/2 pathway in the medullary dorsal horn is closely related to allodynia (Alba-Delgado et al., 2018). Moreover, treatment with PKC inhibitor GF109203X could block the TPA-induced phosphorylation of ERK and JNK proteins, indicating that PKC is an upstream of MAPK family proteins (Chow et al., 2006). These results suggest that the protein expression of p-ERK and NKCC1 is decreased after the application of PKC inhibitor GF109203X, while the protein expression of NKCC1 is decreased after the application of MEK/ERK inhibitor U0126. These results suggest that capsaicin can activate TRPV1 and act on NKCC1 through the PKC/p-ERK signaling pathway. Therefore, after ERK phosphorylation, a part of ERK is placed in the nucleus to play a role in promoting the expression of NKCC1 on the nuclear membrane and the transcription of NKCC1 mRNA, increasing the expression of NKCC1 protein, enhancing the chloride transport capacity, and participating in the regulation of pain mechanism. In addition, the binding site of PKC exists in the molecular structure of NKCC1 (Chamma et al., 2012). PKC not only binds to a large number of intracellular Ca^2+^ molecules but also directly acts on ERK molecules and continues to activate downstream ERK pathway.

The main purpose of this study is to observe the expression and functional changes of downstream NKCC1 transporter by activating TRPV1 and to explore the possible mechanism of the expression and functional changes of NKCC1 in rats with acute inflammatory pain. As shown in Figure 7, increasing the expression and function of NKCC1 in neuronal membrane may promote TRPV1-induced inflammatory pain. The results of this study provide a possibility to focus on the changes and roles of anion channels and transporters in the future research of neuropathic pain and as a new target for the treatment of clinical pain. However, our research also has obvious limitations. For example, although the previous literature was referred to exclude the influence of solvent on the experiment, there was no solvent control group in this study. In this study, the solvents included normal saline and DMSO, but DMSO itself has toxic effects. For example, for the effects of cells and enzymes, more attention should be paid to the control of the concentration of DMSO used, because it is easy to cause cell death. In fact, DMSO is of low toxicity in animals because its LD50 is relatively high. We also referred to previous literatures, so that the concentration of DMSO did not affect the experimental results. Also, in order to better confirm and explain the experimental results, our research needs to refer to the relevant of DRG-NKCC1 knockout or conditional knockdown effective test methods in Dr. Tao’s research group to further confirm these results (Li Z. et al., 2017; Sun et al., 2017; Zhao et al., 2017; Li et al., 2020). In addition, we need to further study whether there is a close relationship between TRPV1 and NKCC1 in molecular structure.

## Conclusion

In conclusion, this study explored a new mechanism of capsaicin activating TRPV1 to mediate the generation and maintenance of inflammatory pain. After injecting exogenous TRPV1 agonist capsaicin into plantar, PKC/p-ERK pathway was activated, which promoted the expression and function of NKCC1 transporter, and increased the intracellular chloride concentration of primary sensory neurons, thus promoting the occurrence of inflammatory pain and plays an important role in pain maintenance.

## Data Availability Statement

The original contributions presented in the study are included in the article, further inquiries can be directed to the corresponding authors.

## Ethics Statement

The animal study was reviewed and approved by the Animal Ethics Committee of the First Affiliated Hospital of Shihezi University School of Medicine (Approval No. A2018-165-01) on February 26, 2018.

## Author Contributions

S-YD and J-QS designed and drafted the manuscript. S-YD, X-CT, Z-ZX, and NC performed the experiments. YW and Q-YC analyzed the data. S-YD drafted the manuscript. Y-CC, K-TM, and LL coordinated and directed the project. Y-CC and J-QS revised the manuscript. All authors read and approved the final manuscript.

## Conflict of Interest

The authors declare that the research was conducted in the absence of any commercial or financial relationships that could be construed as a potential conflict of interest.

## Abbreviations

NKCC1: sodium and potassium chloride co-transporter
DRG: dorsal root ganglion
TRPV1: transient receptor potential vanilloid 1
WB: western blotting
MAQE: N-ethoxycarbonylmethyl -6-methoxyquinolinium bromide
DRR: dorsal root reflex
aCSF: artificial cerebrospinal fluid
PBS: phosphate-buffered saline
TWL: thermal withdrawal latency
CWL: cold withdrawal latency
DMSO: dimethylsulfoxide.
